# Estimation of confidence limits for descriptive indexes derived from autoregressive analysis of time series: Methods and application to heart rate variability

**DOI:** 10.1371/journal.pone.0183230

**Published:** 2017-10-02

**Authors:** Alessandro Beda, David M. Simpson, Luca Faes

**Affiliations:** 1 Department of Electronic Engineering and the Postgraduate Program of Electrical Engineering, Federal University of Minas Gerais, Belo Horizonte, Brazil; 2 Institute of Sound and Vibration Research, University of Southampton, Southampton, United Kingdom; 3 Bruno Kessler Foundation and BIOtech, University of Trento, Trento, Italy; University of Adelaide, AUSTRALIA

## Abstract

The growing interest in personalized medicine requires making inferences from descriptive indexes estimated from individual recordings of physiological signals, with statistical analyses focused on individual differences between/within subjects, rather than comparing supposedly homogeneous cohorts. To this end, methods to compute confidence limits of individual estimates of descriptive indexes are needed. This study introduces numerical methods to compute such confidence limits and perform statistical comparisons between indexes derived from autoregressive (AR) modeling of individual time series. Analytical approaches are generally not viable, because the indexes are usually nonlinear functions of the AR parameters. We exploit Monte Carlo (MC) and Bootstrap (BS) methods to reproduce the sampling distribution of the AR parameters and indexes computed from them. Here, these methods are implemented for spectral and information-theoretic indexes of heart-rate variability (HRV) estimated from AR models of heart-period time series. First, the MS and BC methods are tested in a wide range of synthetic HRV time series, showing good agreement with a gold-standard approach (i.e. multiple realizations of the "true" process driving the simulation). Then, real HRV time series measured from volunteers performing cognitive tasks are considered, documenting (i) the strong variability of confidence limits' width across recordings, (ii) the diversity of individual responses to the same task, and (iii) frequent disagreement between the cohort-average response and that of many individuals. We conclude that MC and BS methods are robust in estimating confidence limits of these AR-based indexes and thus recommended for short-term HRV analysis. Moreover, the strong inter-individual differences in the response to tasks shown by AR-based indexes evidence the need of individual-by-individual assessments of HRV features. Given their generality, MC and BS methods are promising for applications in biomedical signal processing and beyond, providing a powerful new tool for assessing the confidence limits of indexes estimated from individual recordings.

## Introduction

Spectral analysis is a ubiquitous tool for the quantitative analysis of time-series. One example in biomedical applications is the analysis of heart-rate variability (HRV), i.e. the change in intervals between successive heart-beats (heart-period, usually measured as the interval between R waves in the electrocardiogram—thus referred to as R-R intervals). HRV time series typically show a main spectral peak around 0.1 Hz (due to the so-called Mayer waves) and another at a frequency corresponding to the average respiratory rate (due to the so-called respiratory sinus arrhythmia) [1, 2]. From the HRV spectrum, several quantitative indexes can be derived, such as: the total power in the high frequency band (HF, typically 0.15–0.4 Hz), usually associated with parasympathetic (vagal) drive; the power in the low frequencies (LF, typically 0.04–0. 15 Hz), which has been linked (though not exclusively) with sympathetic activity; the ratio between the two, which has been proposed as a marker of sympathovagal balance [3, 4]. Indexes based on information theory, such as entropy or information storage [5, 6], are used to quantify the dynamics of HRV time series [7].

The most common approach in the quantitative assessment of indexes derived from HRV (or other biomedical time series) is to perform statistical analysis to test for significant differences between tasks/treatments/pathological conditions for cohorts of subjects. In this analysis, the focus is on the average behaviour of the cohort rather than that of the individual subjects: the deviation of each individual from the sample average is considered a random effect, and primarily a nuisance, rather than a source of potentially important information. However, in clinical practice, the focus should be on the individual patient being treated/evaluated. With the growing interest in personalized medicine, it is becoming increasingly necessary to be able to reliably assess differences and changes at an individual-by-individual level, not just across a cohort [8, 9]. This requires assessing the accuracy (confidence limits) of the considered descriptive indexes, because their computation suffers from estimation errors due to the random nature of most biomedical time series. The accuracy of estimates may vary across time from measurement to measurement, and also depends on factors such as the length of the series that could be acquired and the (patho)physiological state of the subject. Without some indication of confidence limits/error bounds on estimates, clinical decisions made on individual patients might reflect errors in estimates of otherwise valid indexes, rather than provide a robust assessment of the patient’s underlying condition. The most intuitive choice for finding confidence limits of an index is to repeat the assessment/estimation several times and then to compute the average and dispersion, following a so-called “repeated measures” approach. However this option is not viable in many practical clinical and experimental situations, for reasons that include length of experiment/examination, nonstationarity of the subjects’ response, carryover effects, or irreproducibility of the testing conditions. In these cases, if the single estimated value (point estimate) of the index is above a threshold that separates normality from abnormality, one might wonder if this merely reflects an estimation error or some clinically important dysfunction. Furthermore, if the estimated value for an index changes between two measurements (e.g. after drug administration, or some experimental procedure), can we presume that such a change is statistically/clinically significant, and not just a random effect?

The aim of this paper is to introduce a general framework for the estimation of confidence limits of indexes for individual estimates from single recordings of data. This is a fundamentally different approach compared to the “conventional” one followed in most studies of populations, whereby confidence limits are based on the variability between individuals (i.e., are drawn from the set of values of the index calculated from a cohort of subjects). The framework proposed here is based on a parametric numerical approach. Parametric methods–especially those based on autoregressive (AR) models–have gained ground for spectral analysis as they can provide more robust estimates on short data segments compared to conventional non-parametric methods [10, 11]. Descriptive indexes (e.g., spectral indexes) can then be calculated from the identified model parameters. In this context, when the index of interest can be computed as a linear combination of the model parameters, well-established analytical methods exist to compute its confidence limits (based on the parameters´ estimated values and covariance matrix, both computed as part of the identification process, assuming that their sampling distribution is a multivariate Gaussian distribution) [12]. However, since many indexes are strongly nonlinear functions of the model parameters (e.g., spectral power or entropy), this analytical derivation of the confidence limits is usually mathematically intractable. To overcome this, in this paper we propose and test numerical methods, Monte Carlo (MC) simulations and Bootstrap (BS) surrogate data analysis, which provide powerful and simple means of estimating sampling distributions (see for example [13]) and can be applied to almost any index derived from the estimated model parameters. We show examples of how confidence limits and statistical significance tests can be computed for a given index on an individual-by-individual basis (i.e. based on single time series). We test the effectiveness of the methods on both simulated and recorded time series, and investigate their potential pitfalls. While this paper focuses on a set of indexes typically used in HRV analysis, the general principle can be applied to a much broader range of indexes and to time series beyond the biomedical field.

The Matlab^®^ code implementing the MC and BS algorithms used in this study is freely available for download in the Supporting Information section of this article (S1 File). Sample data used in this study is also made available (S2 File).

## Methods

### Quantitative description of autoregressive processes

Let us consider a stationary stochastic process *X* with zero mean and variance Σ_*X*_. Its autoregressive (AR) representation is defined as

$$x(n)=\overset{p}{\underset{k=1}{\Sigma }}a_{k}x(n-k)+w(n)$$ (1)

where *x*(*n*) is the random variable obtained sampling the process at time *t*_*n*_ *= nT* (*T* is the sampling period), *p* is the order of the process, *a*_*k*_ (*k* = 1, …, *p*) are linear regression coefficients describing the interactions between the process variables as a function of the lag *k*, and *w*(*n*) is generated by an innovation process *W* with variance Σ_*W*_. We consider that the process *X* is fully described by the AR model (1), when the innovation process *W* is formed by independent variables (white noise) which are usually Gaussian. The AR process (1) is then uniquely identified by the parameter set Θ = {**A**,Σ_*W*_}, where **A** = [*a*_*1*_∙∙∙*a*_*p*_]^T^. These parameters can then be used to estimate quantitative indexes describing the process, as outlined below, and will be collectively denoted as *Φ* = *f(*Θ).

The variance of the process (Σ_*X*_) and of the innovations (Σ_*W*_) can be used to derive an information-theoretic description of the statistical structure of the process [5]. For example the information storage *S*_*X*_ quantifies the amount of information carried by *x*(*n*) which can be explained by [*x*(*n*-1), …, *x*(*n*-p)] (i.e. how much of *x*(*n*) can be predicted by its past), and can be computed for an AR process as [5]

$$S_{X}=\frac{1}{2}\text{ln}\frac{\Sigma_{X}}{\Sigma_{W}},$$ (2)

**Fig 1:**
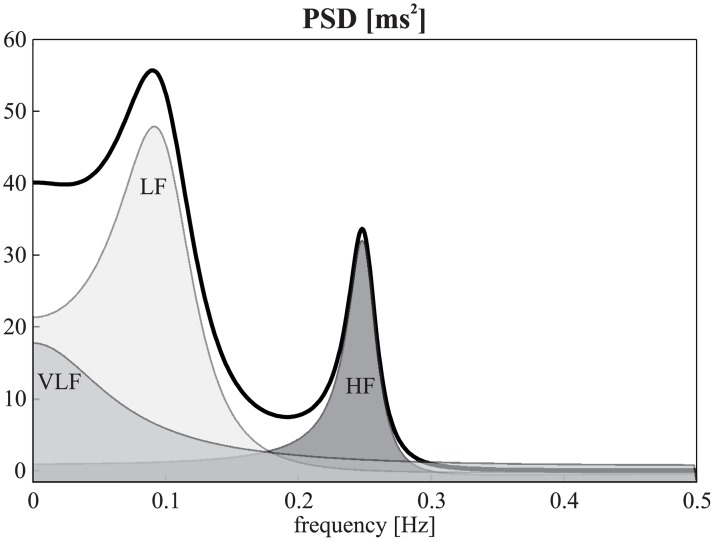
Theoretical spectral profile of the simulated fifth-order AR process and it spectral decomposition. Theoretical spectral profile of a simulated fifth-order AR process of a R-R time series, showing the power spectral density (PSD, thick line) and its decomposition into VLF (very-low frequency), LF and HF spectral components (shaded areas).

In the frequency domain, the power spectral density of the AR process is computed from its coefficients as [14]

$$P(f)=\Sigma_{W}/{\left|A(z){|}_{z=e^{j2\pi fT}}\right|}^{2},$$ (3)

where $A(z)=1-\Sigma_{k=1}^{p}a_{k}z^{-k}$. In this work, we used a spectral decomposition method to split *P*(*f*) into *k* components reflecting the oscillatory structure of the process [15], each associated with a central frequency *f*_*k*_ and a power *P*_*k*_ computed from the roots of the polynomial *A*(*z*) [15]. An example of spectral decomposition is shown in Fig 1.

Among all the possible spectral and information indexes available in the literature based on the AR model, for the sake of simplicity and clarity in this study we only consider an illustrative subset, which is related to HRV analysis: a) the central frequency of the low frequencies peak (*f*_*LF*_, the frequency of the Mayer’s waves [2]), computed as the frequency of the spectral peak nearest to 0.1Hz according to spectral decomposition; b) the LF/HF power ratio (*P*_*LFHF*,_ associated in several studies with sympathovagal balance [1]) computed as the ratio between the spectral power in the LF band and HF band according to spectral decomposition; c) the information storage *S*_*X*_ (inversely related to the complexity of the HRV time series [16]), computed according to Eq (2).

### Estimated sampling distribution for the AR indexes

**Fig 2:**
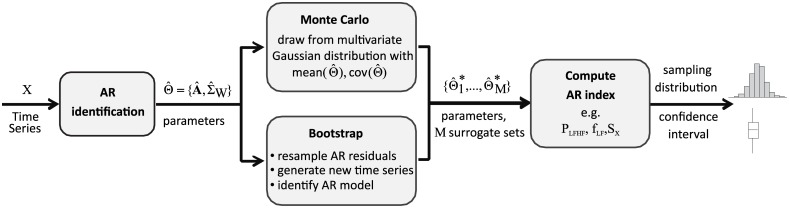
Flow chart of procedure proposed for the estimation of confidence limits. Flow chart of procedure proposed for the estimation of confidence limits of autoregressive indexes descriptive of time series data.

This section describes how to compute confidence limits for indexes estimated from an AR model of an observed process, such as those presented in the previous section. The computation uses either a Monte Carlo (MC) or Bootstrap (BS) resampling approach to estimate the sampling distribution and hence the confidence limits of the indexes. A schematic description of the procedure is reported in Fig 2.

The indexes calculated from the AR representation of the process *X* are estimated from one recording, i.e. from N samples of one realization of the AR process {*x*(1), …, *x*(N)}. The most common estimator follows a least squares approach whereby Eq (1) is evaluated for *n* = *p*+1, …, N and represented in compact form as **X** = **Z**∙**A**+**W**, where **X** = [*x*(*p*+1)⋯*x*(N)]^T^, **W** = [*w*(*p*+1)⋯w(N)]^T^, **A** = [*a*_1_⋯*a*_*p*_]^T^, and **Z** = [**Z**_1_⋯**Z**_*p*_] (with **Z**_*i*_ = [*x*(*p*-*i*+1)⋯*x*(N-*i*)]^T^); then, the coefficients are estimated through the well known least squares formula,

$$\hat{A}={\left[Z^{\text{T}}Z\right]}^{-1}Z^{\text{T}}X,$$ (4)

and the innovation variance is estimated as

$${\hat{\Sigma }}_{W}={\left(\text{N}-p\right)}^{-1}\Sigma_{n=p+1}^{\text{N}}\hat{w}{\left(n\right)}^{2},$$ (5)

with innovations estimated from the residual time series $\hat{W}\text{=}X-Z\hat{A}$.

Assuming that the structure and order of the AR model correspond to those of the process generating x(n), and the estimated innovation is stationary random noise, then the estimated model parameters $\hat{\Theta }=\{\hat{A}\text{,}{\hat{\Sigma }}_{W}\}$ are characterized by a multivariate Gaussian distribution, with mean values given by $\hat{\Theta }$ and variance-covariance matrix for **A** (model coefficients) estimated as [17]

$${\hat{\Sigma }}_{A}={\hat{\Sigma }}_{W}{\left[Z^{\text{T}}Z\right]}^{-1},$$ (6)

and variance for Σ_*W*_ (innovation variance) estimated as

$${\hat{\Sigma }}_{\Sigma_{W}}=\frac{2}{N}{\hat{\Sigma }}_{W}{}^{2}.$$ (7)

Specifically, the use of a multivariate Gaussian distribution is motivated from the sampling distribution of the model parameters, which is usually taken to be a t-distribution, as used in the usual significance tests for regression parameters [18]. When dealing with relatively large sample numbers (say N>100), the t-distribution can be approximated by the Gaussian distribution. According to the central limit theorem, the estimated variance of the innovations is also deemed to follow a Gaussian sampling distribution.

The analytical computation of the distribution (and confidence limits) of derived indexes ($\hat{\Phi }$) is straightforward if these are obtained from a linear combination of AR model parameters ($\hat{\Theta }$). However, this is not possible when the indexes of interest are strongly nonlinear functions of the estimated model parameters $\hat{\Theta }$ (e.g., spectral power, entropy, frequency of a spectral peak). We therefore propose to use numerical rather than analytical means, obtaining the empirical distribution of $\hat{\Theta }$ through MC or BS methods, and hence estimating the empirical distribution (and confidence limits) of the index of interest. It is worth nothing that the proposed approach shares with the analytical approach the same assumptions about the distribution of $\hat{\Theta }$.and validity of AR parameter estimation, and being based on a single realization of the AR process.

The MC approach consists in generating M realizations of multivariate Gaussian noise, with mean values given by $\hat{\Theta }$ and variance-covariance given by Eqs (6) and (7), based on AR model parameter estimates obtained from one recorded data set. The results are M surrogate sets of the model parameters $\hat{\Theta }$, $\left\{{\hat{\Theta }}_{1}^{*},\ldots ,{\hat{\Theta }}_{\text{M}}^{*}\right\}$, and each of these is used to compute a "replication" for each derived index $\left\{{\hat{\Phi }}_{1}^{*},\ldots ,{\hat{\Phi }}_{\text{M}}^{*}\right\}$ (the indexes considered in this study are the information storage *S*_*X*_, the spectral peak frequency *f*_LF_ and the LF/HF power ratio). These M replications form the estimated sampling distribution of the index, which can then be used for computing its confidence limits (see next section).

The BS method takes a different approach and consists of estimating the sampling distribution of the estimates of $\hat{\Theta }$ by first generating M replications of the time series, as described for example in [19], from which M sets of the model parameters $\hat{\Theta }\left\{{\hat{\Theta }}_{1}^{*},\ldots ,{\hat{\Theta }}_{\text{M}}^{*}\right\}$ are obtained through AR model estimation and M replications of the indexes $\left\{{\hat{\Phi }}_{1}^{*},\ldots ,{\hat{\Phi }}_{\text{M}}^{*}\right\}$ are computed. Specifically, the BS replications of the time-series are generated by resampling, with replacement, of the model residuals $\hat{W}=[\hat{w}(p+1),\ldots ,\hat{w}(\text{N})]$, which are sampled with replacement to get a bootstrap sample V=[v(p+1),…,v(N)], where *v*(*i*) is any of the $\hat{w}(i)$ (*i*, *j* = *p*+1, …, *N*). It is important to highlight that bootstrapping has to be applied to the residuals rather than the original time series, since the random resampling will generate white noise, which is appropriate for the residual, but not for the recorded time series. The resampled residuals are then used to generate a new realization of the observed time series, using the equation

$$\tilde{X}=Z\hat{A}+V,$$ (8)

where $\hat{A}$ are the original estimates of the model parameters. From the BS realization a new estimation of model parameters is obtained following the same least-squares approach as in the original estimate

$$\begin{array}{l} \tilde{A}={\left[{\tilde{Z}}^{\text{T}}\tilde{Z}\right]}^{-1}{\tilde{Z}}^{\text{T}}\tilde{X}\\ {\tilde{\Sigma }}_{W}={\left(\text{N}-p\right)}^{-1}\Sigma_{n=p+1}^{\text{N}}\tilde{w}{\left(n\right)}^{2} \end{array},$$ (9)

where the innovations $\hat{w}(n)$ are estimated as $\tilde{W}=\tilde{X}-\tilde{Z}\tilde{A}=\hat{V}$. This procedure is repeated M times, resulting in M replications of $\hat{\Theta }$, $\left\{{\hat{\Theta }}_{1}^{*},\ldots ,{\hat{\Theta }}_{\text{M}}^{*}\right\}$ which are then used to compute confidence limits for spectral, information theory, and other indexes in the same way described for the MC method above.

### Comparison and statistical testing of difference between two individual estimates

The MC or the BC procedure outlined in the previous section generates an estimate of the sampling distribution of the index, which will be denominated as the empirical distribution. This can be used to compute confidence limits for the estimate, based on the appropriate percentiles of this distribution. Throughout the paper, confidence limits for the estimates of these indexes are computed and shown as the 25^th^-75^th^ (interquartile) or 5^th^-95^th^ percentile intervals of the distribution.

In addition, the estimated empirical distributions can be used to test for the existence of a statistically significant difference between estimates (say S1 and S2) of a given AR-derived index on an individual-by-individual basis. This allows using the MC or BC procedure for hypothesis testing in order to assess, for example, the difference between two subjects, or between two treatments or tasks in the same subject, based on single pairs of recordings. In this work, we propose one possible approach for such tests. First, the empirical distribution of the difference S2-S1 is computed by subtracting the M sample estimates of S1 from the M sample estimates of S2 (M = 1000 in this study), with both obtained using either the BS or the MC method. The order in which the M samples are chosen should be random; we did not perform the subtraction of all possible MxM samples combinations, because the resulting empirical distribution would not have been of independent samples and computational load excessive. Then the null hypothesis that the difference between S1 and S2 is zero is rejected with a significant level α, if zero is outside the interval between the 100∙α/2 percentile and the 100∙(1-α/2) percentile (for a two-sided test) of the empirical distribution of S2-S1. Note that the test relies on the key assumptions that the data follows a stationary AR model, and the model order is appropriate—the same assumption as made in most statistical analyses of AR models. Examples will be presented below to illustrate these tests. In this work, all tests are deemed significant at α = 0.05.

## Assessment of methods using simulated data

While the basic principles of the MC and BS methods are firmly established in the literature, they have not, to the best of our knowledge, been applied and tested in the assessment of HRV indexes, or similar measures derived from AR spectral estimates in other applications. Therefore, in this section we will test them first in well-controlled simulated time series. The simulation study is conceived to explore the estimation of confidence limits of AR-derived indexes in a situation in which: (i) the theoretical values of the AR model parameters are known *a priori*; and (ii) the “true” empirical distribution and confidence limits for any index can be derived empirically through multiple realizations of the known AR process. These values can then be compared to those obtained from an individual realization using the MC and BS methods, to confirm that the latter provide robust estimates of the former. The performance of the BC and MS methods was assessed by varying the length of the time series, spectral content of the AR process, and assumed model order. Then, it was further tested under some conditions of nonstationarity and nonlinearity of the HRV dynamics, simulated respectively by superimposing sinusoidal trends to the AR time series and introducing nonlinear or regime-switching properties in the model equations. While such simulations cannot be comprehensive and don’t allow generalization to all possible cases, they can provide an indication of the sensitivity of the results to the assumption of stationarity.

### Simulation design

The simulation was aimed at reproducing the oscillatory activity typically observed in short-term R-R time series, including LF, HF, and very low frequency (VLF, 0–0.04 Hz) components [10]. Specifically, we considered a fifth order AR process with model parameters set to reproduce a typical HRV spectral profile with peaks at LF (~0.1 Hz) and HF (~0.25 Hz) superimposed on very slow fluctuations with a spectrum slowly decreasing with frequency, thus emulating R-R time series with an average heart period of 1s. To this end, the model parameters were chosen in order to obtain a transfer function with two complex-conjugate poles with modulus ρ_LF_ = 0.8 and phases ±2π*f*_LF_ (*f*_LF_ = 0.1), two other complex-conjugate poles with modulus ρ_HF_ = 0.92 and phases ±2π*f*_HF_ (*f*_HF_ = 0.25), and a real pole with modulus ρ_VLF_ = 0.65 (*f*_VLF_ = 0). The corresponding AR parameters are: *a*_*1*_ = 1.944, *a*_*2*_ = -2.238, *a*_*3*_ = 2.062, *a*_*4*_ = 1.254, *a*_*5*_ = 0.352. Gaussian innovations were set to variance Σ_*W*_ = 1. The theoretical spectral profile of the simulated process is shown in Fig 1. Given this representative setting for the AR parameters, the simulation study was designed to generate realizations of the AR process (by feeding the AR model with realizations of white noise with zero mean and variance Σ_*W*_) consisting of 300 samples (where not indicated otherwise), which match those typically used in R-R time series, where 5 minutes of data are recommended [10]. For each realization, we estimated the indexes *f*_LF_, *P*_LFHF_, *S*_*X*_, their empirical distribution through the BS and MC approach, and the confidence limits of the estimates using the methods described in details in the “Methods” section. Moreover, these confidence limits were compared with the confidence limits of the "true" empirical distribution of each index obtained from computing the index from multiple realizations (1000 in this study) of the AR process. This distribution, which is considered here as a "gold standard" for the comparison, can only be obtained in simulations where the true values of the model parameters are known and can be used to replicate the process.

Then, we assessed how the estimates of the indexes *f*_LF_, *P*_LFHF_, *S*_*X*_ are affected by changes in parameters such as the time series length (120, 300, 600 samples), the modulus and frequency of the pole associated with the LF oscillation (0.6, 0.7, 08, 0.9 a.u. and 0.05, 0.1, 015 Hz, respectively), and the model order used for the AR parameters estimation (3, 5, 7, 9, while the model order was always 5 for all simulations). Also, we investigated the effects of deviations from the assumption of a linear stationary time series by simulating nonstationary and nonlinear behaviours as follows. Nonstationarities were induced by adding to the original time series a sine wave of period *T* and amplitude *M*. Specifically, we studied the dependence on the period and on the amplitude of the sinusoidal trend respectively by varying *T* in the range (0, 300, 600, 1200 sec) with *M* = $\sqrt{\Sigma_{X}}$, and then by varying *M* in the range (0%, 50%, 100%, 150%) of $\sqrt{\Sigma_{X}}$ with *T* = 600. Furthermore, nonlinearities were introduced in two different ways. Squared nonlinear dynamics were obtained modifying Eq (1) with the addition of quadratic terms:

$$x(n)=\Sigma_{k=1}^{5}a_{k}x(n-k)+\beta (x^{2}(n-1)-x^{2}(n-2))+w(n),$$ (10)

where an increasing importance of squared nonlinearities was obtained varying β in the range (0, 0.02, 0.04, 0.06). Alternatively, threshold AR processes were simulated switching between two linear regimes, as:

$$x(n)=\{\begin{array}{lll} \overset{5}{\underset{k=1}{\Sigma }}a_{k}x(n-k)+w(n) & \text{if} & x(n-1)\leq \gamma \\ \overset{5}{\underset{k=1}{\Sigma }}b_{k}x(n-k)+w(n) & \text{if} & x(n-1)>\gamma \end{array},$$ (11)

where *a*_*k*_ are the same used in the other simulations, and *b*_*k*_ were chosen to obtain a more irregular and slow LF spectral component (ρ_LF_ = 0.4 and *f*_LF_ = 0.07, resulting in *b*_*1*_ = 1.363, *b*_*2*_ = -1.470, *b*_*3*_ = 1.257, *b*_*4*_ = -0.528, *b*_*5*_ = 0.088). An increasing presence of the second regime was obtained decreasing γ in the range (300%, 180%, 140%, 100%) of Σ_*X*_.

### Statistical analysis

For each combination of parameters considered, we simulated 100 realizations of the AR process. Then, for each realization, we computed the confidence limits of each index using the BS and MC methods, and the statistical test described at the end of the “Methods” section was applied to assess whether *f*_*LF*_, *P*_*LFHF*_, and *S*_*X*_ changed significantly when a parameter was varied. Then, the number of times (out of 100) for which such test detected a statistically significant change was recorded. The average BS and MC confidence limits (i.e. average 5^th^, 25^th^, 75^th^, 95^th^ percentiles) were compared with those yielded by the “gold standard” approach of generating 100 realizations of the original process.

### Results

**Fig 3:**
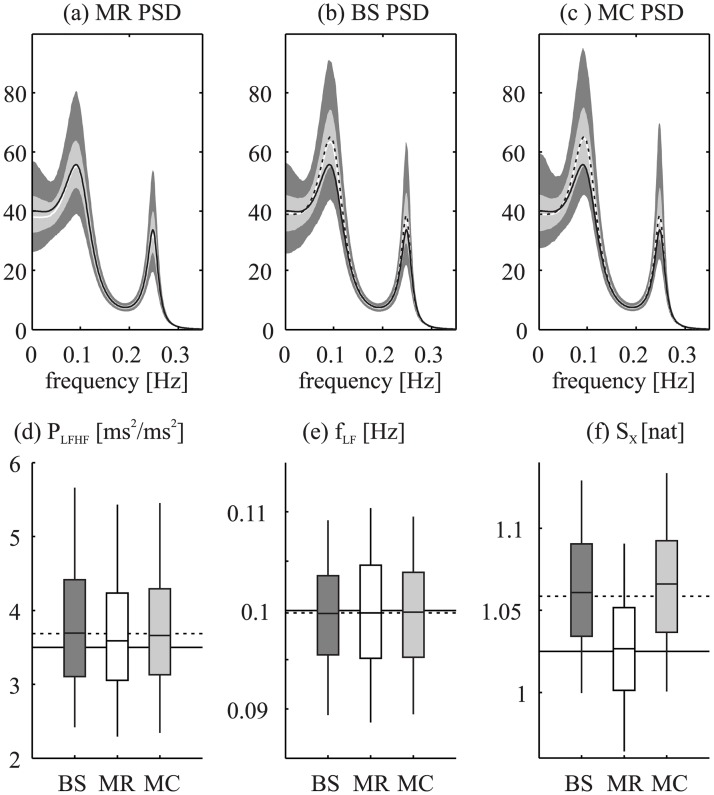
Confidence limits estimation of spectral and information indexes for the simulated AR process. Representative example of the estimation of confidence limits of spectral and information indexes for the simulated AR process (typical parameter setting) using the multiple realizations ("gold standard"), or a single realization with the BS or MC. Upper plots show the sampling distribution of power spectral density assessed from: (a) “gold standard”; (b) one realization using BS; (c) one realization using MC; darker area 5–95% interval, lighter area 25–75% interval, white line median, dashed black line estimate, solid black line true value (based on known AR model parameters). Lower plots show confidence limits of (d) LF/HF power ratio (P_LFHF_), (e) frequency of the LF component (f_LF_), (f) and information storage (S_X_), obtained using the “gold standard” (white box plot), a single realization with BS (dark gray) and a single realization with MC (light gray). In the confidence limits plots, the box represents the 25–75% range (the horizontal line in the middle is the median) and the vertical lines the 5–95% range; for each index, the plots report also the true value as obtained from the selected AR parameters (solid horizontal line) and the value estimated for the single realization used to assess both BS and MC confidence limits (dotted horizontal line). Note that the MC and BS methods simulate one random realization rather than the original process (which is not available when using these methods), so one should not expect the mean of the distributions to agree, but they should be within the confidence limits. Note that the widths of the error bars are similar in all cases.

Fig 3 illustrates the results of the analysis performed on a representative example of the model parameters. For this configuration, the computational times relevant to the calculation of the confidence limits of the AR parameters on a single realization of the simulation were equal to ~1 millisecond using the MC method, and to ~2.15 seconds using the BS method, respectively, when running a Matlab^®^ script on an off-the-shelf computer (Intel Core Duo CPU, 2.66 GHz) under Windows^®^. Fig 3a reports the sampling distribution of the spectral profiles obtained by generating multiple realizations of the original process with the chosen model parameters. The sampling distribution over multiple realizations, taken here as “gold standard”, is centred around the true spectral density (shown as black solid line), though with some small bias due to the fact that the estimated spectra are nonlinear functions of the estimated coefficients. Fig 3b and 3c report the sampling distributions of the spectral functions estimated from one (random) realization of the original process (spectrum shown as a dashed line), followed by the BS and MC approaches. Again there is some bias in the BS and MC estimates (the dashed and the white line do not fully agree), but they are well within the confidence limits. A more evident bias is the deviation of the estimated spectrum and its associated MC and BS simulations from the true profile (i.e. the difference between the dashed and solid black lines), which is expected and unavoidable, since the former is an estimate of the latter, using one realization of the process. Notwithstanding this, the true value is always found within the 25–75% confidence limits of the estimated value. Similar results and interpretations are found for the assessment of the sampling distributions of the indexes derived from the AR model (i.e., *f*_LF_, *P*_LFHF_, and *S*_*X*_, Fig 3d, 3e and 3f): the values obtained from multiple realizations are distributed around the true value, and the values obtained from BS or MC are distributed around the estimated value. An important observation is that the confidence limits obtained through the three approaches exhibit comparable widths for all indexes.

**Fig 4:**
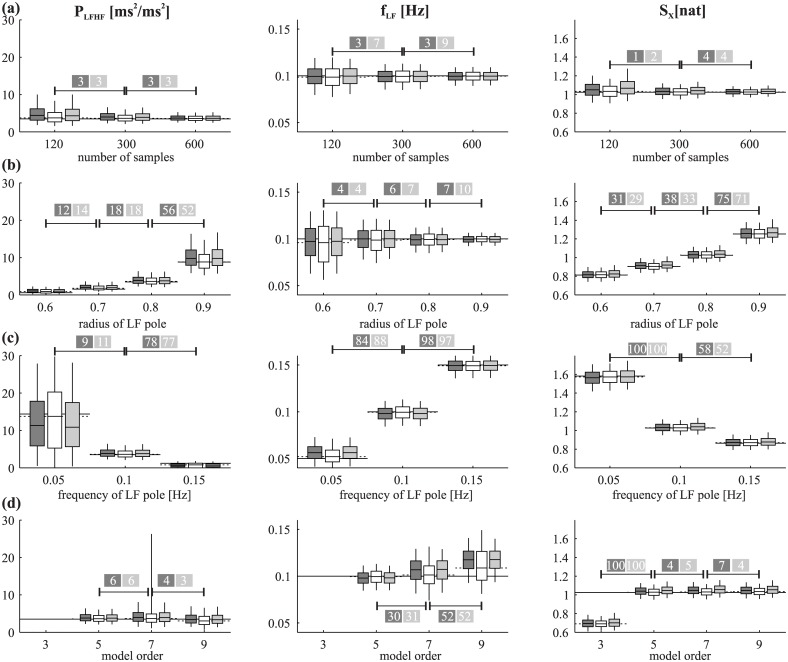
Assessment of methods for confidence limits estimation at varying simulation parameters. Assessment of methods at varying simulation parameters: (a) time series length; (b) radius of the pole of the LF oscillation; (c) frequency of the LF oscillation; (d) order of AR model used for estimation. Plots depict average confidence limits (mean over 100 simulation runs of the values of 5^th^, 25^th^, 50^th^, 75^th^, 95^th^ percentiles) of the LF/HF power ratio (P_LFHF_), frequency of the LF component (f_LF_) and information storage (S_X_) of R-R intervals assessed from multiple realizations (white, the “gold standard”), from a single realization using BS (dark grey), and from a single realization using MC (light grey). In the confidence limits plot the box represents the 25–75% range (the horizontal line in the middle is the median) and the vertical lines the 5–95% range; for each index, the plots report also the true value (solid horizontal line) and the average value estimated for the single realization used to assess BS and MC confidence limits (dotted horizontal line). The numbers in the coloured boxes represent in how many cases out of 100 a significant change between consecutive value of the parameters was detected using the test described at the end of the “Methods” section, considering either the MC (dark grey box) or BS (light grey box) approach.

**Fig 5:**
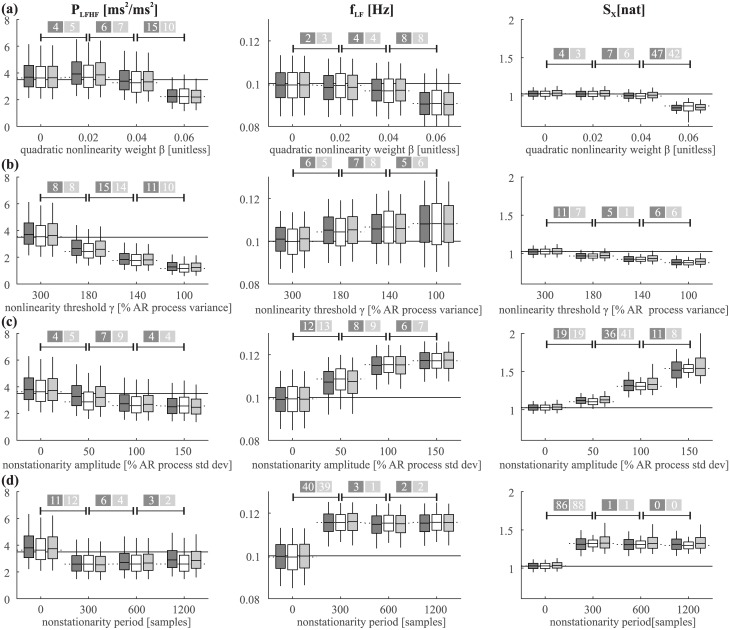
Assessment of methods for confidence limits estimation in presence of nonlinearity or nonstationarity. Nonlinearities were introduced as: (a) Squared nonlinear dynamics of increasing magnitude, see Eq (10); (b) switching between two nonlinear regimes with increasing recurrence, see Eq (11). Nonstationarities were introduced as: (c) a sinusoidal trend with increasing amplitude; (d) a sinusoidal trend with increasing period. Confidence limits, colours, and symbols are the same used in Fig 4.

Figs 4 and 5 report the assessment of methods when varying the model/simulation parameters or introducing nonlinearities and nonsationarities, showing how such changes resulted in alterations in the true and estimated values of each index, and in the width of the confidence limits. Overall, the two figures show that the confidence limits obtained with the three methods (multiple realizations, MC and BS) are very similar in almost all cases. Their width changes in agreement with each other between simulations, in accordance with what might be expected.

The dependence on the time series length is illustrated in Fig 4a. As expected, the confidence limits reduced in size with increasing length (p<0.02 when performing a Student t-test comparing the confidence limits size between N = 120 and N = 600, for all indexes) while the bias remained well within interquartile range in all cases. Also, the chance of detecting a significant change in the parameters from a single acquisition only by increasing the length of the time series was below 10 times out of 100 simulations, which is within the expected range when there is no change in mean value (as one would expect in this case). With the false positive rate of 5% used in this work, in 100 simulations one would expect (with α = 0.95) up to 9 false positives (according to the binomial distribution with p = 0.05 and 100 trials).

Fig 4b illustrates the dependence of the indexes on the radius of the pole identifying the LF oscillation. Increasing the radius results in larger LF oscillation (higher *P*_LFHF_) without changes in its central frequency (no change in *f*_LF_); as the signal becomes more strongly oscillatory at LF, it is also characterized by “simpler” overall dynamics (higher *S*_*X*_). The likelihood of detecting a significant difference from single time series was high (i.e. above 50%) only when comparing the highest radius value considered (0.9) with the others.

Fig 4c illustrates the effect of changes in the LF central frequency (*f*_LF_). These changes in *f*_LF_ were reliably detected by all methods (successful detection of change in 84 simulations or more out of 100). Furthermore the decrease in *P*_LFHF_ and *S*_*X*_ was expected by the fact that the central band was moving from the lower limit to beyond the upper limit of the LF range. The difference in *P*_LFHF_ between *f*_LF_ = 0.05Hz and 0.1Hz is a typical example of poor detection of a significant difference from single time series (successful detection in 11 simulation or less out of 100), when average difference is considerable but confidence limits are wide.

Fig 4d shows the analysis performed by varying the order of the AR model used for identification, while using the same 5^th^ order model for the simulations. While “overfitting” (i.e. using a model order larger than the true value) did not greatly affect the estimated parameters and their confidence limits, identifying a model order below the true value (i.e. 3 in this case) generated very discrepant results: *f*_LF_ and *P*_LFHF_ and their confidence limits could not be computed, because the spectral decomposition produced only one component located in the HF band; *S*_*X*_ was significantly lower (i.e. differences between random pairs of recordings with different estimation model orders were detected in 100% of cases).

Fig 5a and 5b show that the presence of mild nonlinearities (i.e. β = 0.02 in Fig 5a or γ = 300% if Fig 5b) does not greatly affect the appearance and reliability of the confidence limits. Expectedly, as nonlinearities become more pronounced, the simulated process is progressively less comparable to the "original" linear AR process, and consequently the estimated values tend to shift away from the values obtained for the purely linear process. Notably, this effect is found not only for MC and BS, but also for the "gold standard" approach (multiple realizations), indicating that nonlinearities would alter the value of each index also in the ideal condition of having infinite realizations of the process. In addition, we found that pronounced nonlinearities not necessarily result in larger confidence limits, as in most cases their width was comparable (or even smaller) when increasing the degree of nonlinearity. Furthermore, the statistical tests shown in the figures suggest that moderate increase/decrease of the magnitude of nonlinearities are unlikely to result in the detection of a significant change in the indexes considered. Similar considerations can be made regarding the results shown in Fig 5c and 5d about nonstationarities. Superimposed trends of small magnitude (50% of the AR process standard deviation., Fig 5c) do not affect substantially the estimated values, but as the magnitude increases, the indexes shift away from the values obtained for the purely linear process, apparently independently of the periodicity of such trends (Fig 5d). Again, the result is quite expected since the simulated process become progressively less compatible with the original linear AR process as the degree of nonstationarity increases. It may again be noted that in almost all cases the confidence limits for all three methods (MC, BS and the “gold standard” using multiple realizations) are similar, for all indexes and simulations.

## Assessment of methods using data recorded from human volunteers

### Experimental protocol

In the experimental dataset considered for this study, described in details elsewhere [20], 25 subjects (mean age (SD) 26.5 (4.0), 12 women) undertook, among others, the following tasks: baseline rest—relaxed and without talking; math silent—performing a series of subtractions (repeatedly subtracting seven from three-digit random numbers) presented on sheets on which also the answers had to be written; math aloud—same as math silent, but having to read all the questions and answers aloud. The tasks each had 5 minutes duration and were separated by rest periods of 5–7 minutes. ECG was monitored continuously with a sampling frequency of 1000 Hz, and the R-R time series was extracted from the ECG using an automated algorithm followed by manual editing [20].

The indexes *f*_LF_, *P*_LFHF_, and *S*_*X*_ were computed as described in the “Methods” section for each subject in each task, as well as their average values. Confidence limits were estimated for each individual recording using the BS and MC methods. The order of the AR model was chosen automatically for each subject/task in the range 5–15 using the Akaike criterion [14].

### Statistical analysis

The statistical significance of the change between tasks was assessed for each individual using the method described at the end of the “Methods” section. The results were compared with more conventional cohort-based analyses using the Wilcoxon signed rank test, with a significance level α = 0.05.

### Results

**Fig 6:**
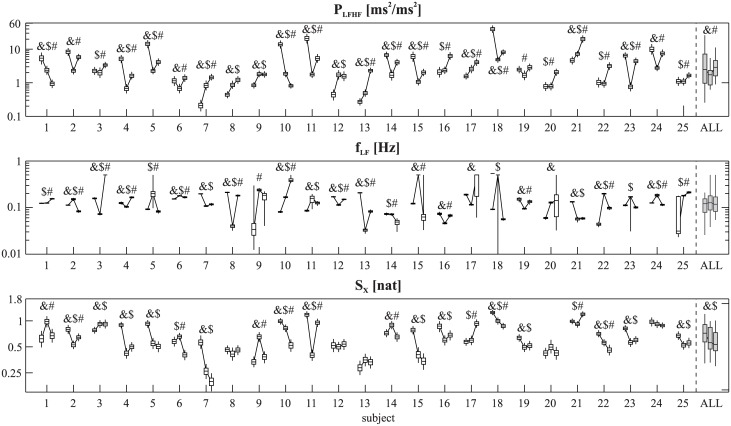
Individual-by-individual evaluation of changes across conditions based on MC confidence limits for the experimental dataset. Confidence limits obtained for LF/HF power ratio (P_LFHF_), frequency of the LF component (f_LF_), and information storage (S_X_) of R-R intervals, are shown for each of the 25 subjects during three tasks (rest, math silent, math aloud, from left to right). Confidence limits for the whole sample are also shown in grey on the right (ALL). In the confidence limits plots, the box represents the 25–75% range (the horizontal line in the middle is the median) and the vertical lines the 5–95% range; Symbols indicate statistically significant difference between tasks (computed as described at the end of the “Methods” section) for individuals estimates 1 to 25 and Wilcoxon sign-rank test for the whole sample, p<0.05): &, rest vs. math silent; $, rest vs. math aloud; #, math silent vs. math aloud. Note the large diversity between individuals in the direction and magnitude of response, and in confidence limits width.

Fig 6 shows, for each subject and each protocol, the estimate of each index, together with their confidence limits obtained using the MC method. As already expected from the simulation studies, results using BS were very similar and thus are not reproduced here. It is evident that different subjects showed different patterns of change between tasks, and that those changes are significant at an individual level. It was surprisingly rare that individuals showed a similar pattern of change to that of sample average (“ALL” in Fig 6). For example, for *P*_LFHF_, five subjects (7, 8, 13, 17, 21) showed a consistent pattern of increase from rest to math silent to math aloud, while other subjects showed completely different patterns (e.g. the opposite trend in subjects 1 and 10). What the estimated confidence limits and statistical tests now show is that not only is the pattern different, but also that the differences cannot be explained simply by random estimation errors. The width of confidence limits also varied considerably between subjects and tasks; some recordings clearly provide more robust estimates than others. The width of the confidence limits for *f*_*LF*_ showed the most dramatic variations across subjects and tasks (e.g. subject 9): the very wide confidence limits indicate the absence of a consistent spectral peak in the LF band and thus that the *f*_*LF*_ estimates are not reliable in many cases.

Table 1 compares the results of statistically testing the significance of changes between tasks with a conventional test (Wilcoxon signed-rank test) with the results obtained using our MC method based on differences in each individual subject (results for BS were very similar, not shown). The conventional group-wise analysis of the changes across conditions indicates that: (i) *P*_LFHF_ is significantly lower in maths silent than the other tasks; (ii) *f*_LF_ did not change significantly across tasks; and (iii) *S*_*X*_ is significantly higher at rest than in the other tasks. The corresponding analysis performed by the MC method confirms that only some individuals follow the group-wise pattern, suggesting that sample-average trends are the combination of heterogeneous individual responses rather than the repetition of a coherent response with added random effects due to parameter estimation errors. For instance, while according to group wise analysis *P*_LFHF_ decreases significantly from rest to math silent, according to the MC method a significant decrease occurred only in 44% of the individual subjects, with the rest significantly increasing (24% of subjects) or not showing a significant change (32%). The significant decrease in group-wise median of *S*_*X*_ from rest to math silent or math aloud is also only followed by less than 50% of individuals according to the individual MC analysis, with the reminder showing no significant change (48% or more) or even an increase (4%). For *P*_LFHF_, on the other hand, the increase between math silent and math aloud is followed by a clear majority of individuals (72%), and similarly for *S*_*X*_ the majority follow the non-significant change in median value. These results clearly justify the need for the individual confidence limits and statistical tests, as proposed in this paper, to provide a clearer insight of the behaviour of the individual within the context of the cohort he/she belongs to. This is important in order to avoid the misleading inference that all, or even a majority, necessarily follow group average behaviour and that random estimation errors can explain deviations of individuals from group-average behaviour, and can thus be disregarded.

**Table 1:** Statistical test of changes between experimental tasks.

	sample-average approach (Wilcoxon signed rank)		Individual approach (Monte Carlo method)		
	difference (irq)	p-value	%increase	%ns	%decrease
P_LFHF_ [ms^2^/ ms^2^]					
rest→maths	-0.47 (6.10)	0.020	24	32	44
rest→matha	0.40 (4.92)	0.288	40	32	28
maths→matha	1.41 (2.76)	0.000	72	20	8
f_LF_ [Hz]					
rest→maths	0.03 (0.15)	0.353	32	32	36
rest→matha	-0.01 (0.07)	0.493	20	48	32
maths→matha	0.00 (0.11)	0.861	36	48	16
S_X_ [nats]					
rest→maths	-0.15 (0.34)	0.040	4	52	44
rest→matha	-0.12 (0.30)	0.009	4	48	48
maths→matha	-0.02 (0.16)	0.619	12	72	16

R: rest; MS: math silent; MA: math aloud; irq: interquartile range; %increase, %decrease, %ns: percentage of the subjects for which a significant increase, significant decrease, or non-significant change was identified using the MC methods from the single recordings.

## Discussion

We have introduced an empirical/numerical framework to calculate confidence limits of a descriptive index derived from individual realizations of a stochastic AR process. The framework makes use of the computationally intensive statistical methods of MC and BS, through which the empirical sampling distribution of the index is built up to extract the confidence limits and assess statistical significance. In this study, the framework is tested with reference to AR-derived indexes based on spectral power, spectral frequency and entropy/predictability, and is validated using both simulated processes and recorded HRV time series measured during psychophysiological tasks.

The proposed framework is novel in several respects. First, it provides a unique approach that could be used to estimate confidence limits of any index which is a function of the AR coefficients, independently of such function being linear or not. This is a considerable advantage to conventional analytical methods for estimating confidence limits, which require specific mathematical derivations for each index and make (often unreliable) assumptions about the shape of its sample distribution (e.g., Gaussianity). Second, while the use of MC and BS methods for estimating confidence limits is well established [21, 22], we are not aware of previous reports formulating and testing their application as proposed in this paper, i.e. deriving confidence limits of nonlinear functions of parametric model coefficients estimated from individual time series, for within- and between- individuals statistical comparison, as shown for example in Fig 6. The software code we provide in the Supporting Information section of the article will allow researchers to perform the same individual-based analysis proposed in this paper using any index they might be interested in (as long as it is derived from AR model coefficients). Third, the proposed framework offers an approach for the estimation of confidence limits of indexes that is different to the conventional approach of repeated measures [23,24]. In the latter, several realizations of the time series are needed to estimate the index and then its confidence limits form the estimated mean and dispersion, while in the proposed approach only one realization is considered. The two approaches are complementary but not equivalent, and the proposed one is especially relevant when repeated measures are not available, which is the case in many practical, clinical, and experimental scenarios.

### Robustness of the computation of confidence limits from single realization of AR processes

Our results suggest that the proposed framework is robust for the AR analysis of R-R time series, and thus promising for related applications. This was documented by the good agreement between the MC and BS methods and the “gold standard” of multiple realizations in the simulations, by the fact that confidence limits get wider as time series become shorter (Fig 4a), and by other results that agree with the changes in confidence limits that one might expect (see below).

Our simulation results document that the BS and MC methods applied to single time series produce similar confidence limits in all cases, and that these limits have a width comparable to that of the “gold standard” intervals obtained through multiple realizations of the “true” underlying process. This provides additional confidence that BS and MC give robust results in this application. Moreover, the statistical tests we performed show that BS and MC can be used to reliably detect a statistically significant change in the indexes when such a change is expected (modification of the AR parameters of the process), as well as the absence of significant changes when the index is expected to remain stable, based on a single realization of the process. Clear examples of the agreement between multiple realizations and BS or MC resampling schemes are the increase of *P*_*LFHF*_ and *S*_*X*_ obtained at increasing the radius of the LF pole (Fig 4b) or at decreasing the frequency of the LF component (Fig 4c), the invariance of all indexes across changes in the length of the observed realizations (Fig 4a), and their alteration induced by mismatches between the order of the simulated and estimated process (Fig 4d). Also the presence of mild nonstationary or nonlinear behaviors was found not to alter the width of the confidence limits or the agreement between multiple realizations and MS/BC (Fig 5). Notably, this agreement stands in most cases also when the confidence limits are clearly biased. This means that also in the ideal condition of having multiple realizations of the process (i.e. our "gold standard" approach) the bias would still exists, indicating that it is not related to the estimation process of the confidence limits, but rather to the discrepancy between the model used for identification and the true process generating the time series. This issue is further addressed in the "Limitations" section.

### Comparison of MC and BS methods for the computation of confidence limits

The fact that the MC and BS methods exhibited comparable performance speaks in favour of the utilization of the MC method, which has significantly lower computational costs. In fact, the BS approach requires a least squares estimation of the AR parameters for each of the M simulated time series, whereas the MC approach only requires generating M sets of the AR parameters based on a multivariate distribution whose mean and covariance matrix are estimated only once using a least square approach. However, the MC method is based on the assumption of Gaussianity of the distribution of the AR model coefficients, while the BS is not (since it is based on resampling the residuals regardless of their amplitude distribution). Such assumption can be justified based on the central limit theorem and when using fairly large samples (section 9.6 in [12]). We have tested performance down to 120 samples (120 seconds), with good results, but would expect performance to degrade for very short segments. In short samples also the estimates of covariances are expected to become less robust, and with it would the estimates of confidence limits and significant differences. Hence, the BS method may be more appropriate in the case of very short recordings or non-Gaussian residuals. The latter cases have been found useful, for example in the representation of causal interactions in multivariate settings [25].

### Estimation of confidence limits of AR-based indexes in heart rate variability analysis

The application of the framework to the HRV time series of the psychophysiological protocol considered in this study reveals that the variation of HRV indexes during conditions of mental stress is highly heterogeneous, with different subjects displaying different patterns of change between conditions. In quite a few cases there is clear disagreement between the average behaviour of the sample (see ALL in Fig 6), which reflects the way such data is usually looked at, and that of individuals. Furthermore, the results from our MC and BS simulations show that in many cases this disagreement cannot be explained by random estimation errors but presumably reflects genuine differences between individuals (Table 1), or differences which may reflect the physiological status of that subject during that experiment, which could change with repeated recordings. For instance, the statistically significant sample average decrease of *P*_*LFHF*_ observed from rest to math silent was found also at an individual level in only 44% of the subjects by the MC method, while 24% of the subjects showed a significant increase. Similarly, the significant group-wise decrease of *S*_*X*_ when moving from rest to math silent or math aloud could only be detected on an individual basis in less than half of the subjects. The *P*_LFHF_ and *S*_*X*_ are measures reflecting the neural regulation of HRV, respectively in terms of sympatho-vagal balance [1] and dynamic complexity [26]. In agreement with previous studies, our results suggest that the patterns of alterations in the neuro-autonomic activity induced on HRV by mental stress are more complex and inter-individually variable than those elicited by other types of physiological stress, such as orthostatism [6, 27, 28].

On the other hand, sample average and individual-based approaches led to similar results when assessing the change from math silent to math aloud, indicating an absence of significant changes of *S*_*X*_, as well as a significant increase of *P*_LFHF_ (Table 1). The latter finding is in agreement with a previous investigation using the same dataset [20], which showed that tasks involving speech (e.g. math aloud) elicited an increase of respiratory oscillations within the LF band, which is expected to induce an increase in LF power of HRV through parasympathetic neural pathways [29]. More generally, the high inter-individual variability of the descriptive indexes of HRV observed in the present and other psychophysiological datasets [30, 31] is likely related to the fact that for the tasks considered (e.g. cognitive/behavioural challenges), and for real-life situations in general, it is impossible to tightly control the experimental conditions. This can evoke highly erratic responses (including strong respiratory responses) which are not consistent across subjects [20], and may thus result in wide diversity in HRV changes. In situations like this, and in any experiment in which consistent and/or reproducible responses are not expected, it is therefore strongly recommended to investigate individual responses rather than merely studying the average behaviour of a group and sample average trends across different physiological states or experimental conditions.

### Limitations: Confidence limits (computed analytically or numerically) are not a “magic bullet”

A fundamental assumption for the estimation of confidence limits of indexes computed from AR model parameters is that the measured time series is a realization of a linear stationary Gaussian process. This assumption must hold for analytical and numerical approaches for confidence limits estimation; when it is violated, the reliability of inferences based on confidence limits is affected, independently of the method adopted. In this work we explored the effects of some of such violations, by studying the behaviour of the AR descriptive indexes and of their confidence limits when nonstationary and nonlinearity are introduced in the processes. Our results suggest that the MC and BS methods are robust to mild violations of the assumption of stationarity and linearity. Indeed, increasing the degree of nonstationary or nonlinear behaviors imposed in the simulated processes did not alter significantly the width of the confidence limits, which remained well centered around the estimated value of the descriptive index to which they refer (Fig 5). Nevertheless, as nonstationary or nonlinear behaviors increase, the bias grows between the estimated indexes and their value obtained from the "original" linear AR process, but this is the result of violation of the linearity and stationarity assumptions, and cannot be “accounted for” by any method (numerical or analytical) of confidence interval estimation based on such assumption. Clearly, a comprehensive analysis of the magnitude and types of violation of the validity of model identification is impossible. Hence, in general terms, confidence limits (analytically or numerically computed) are not a “magic bullet” to compensate or account for poor model identification, and a thorough validation of the identification process (proper model structure and order selection, testing for randomness of residuals) is always necessary.

Another fundamental underlying assumption for the confidence limits estimation of an index is that the estimator used is unbiased. When this assumption is violated, the possibility arises that, due to the bias, the true value of the index might lie outside the X% confidence limit in more that 100-X% of cases. This issue is not specific to the BS and MC methods presented in this work, but is common to all methods of confidence limits estimation (including analytical), and is recurrent when dealing with spectral indexes. For example, it is trivial to show that entropy, or the spectral power for a given frequency (or frequency band), estimated from the coefficients of and AR model, are biased estimators. Hence, it is not surprising that, for some indexes, the BS and MC methods can result in confidence limits that are different from those obtained by the “gold standard” (multiple realizations). As exemplified in Fig 3f for the entropy-based measure, the width of the confidence limits is similar, but the sampling distribution for BS and MC is centred around the estimate of the parameter, while for the “gold standard” is centred around the true value of the parameter—which is typically only known for simulation studies and is not known in most applications where confidence limits are most needed. This difference of course raises concern regarding the capability of BS and MC to detect when significant changes in the parameters are present. It is not possible to formally demonstrate the contrary or to generalize for all conceivable indexes of HRV or other signals, but in our results the level of this bias was generally quite small compared with the amplitude of the confidence limits. Nevertheless, the effect of the combination of bias in the estimator and error in AR coefficients estimation is difficult to quantify, and possibly should be assessed for the specific application of interest (i.e. when time series characteristics, model structure, and index of interest are known).

### Future developments and research

First, we expect to extend the MC and BS methods AR models to ARX models (with single or multiple inputs) and their nonlinear counterparts, in order to broaden the scope of the methods and to estimate confidence limits of relevant features (e.g. indexes) of transfer functions of linear models, such as gain and phase for specific frequencies or average values over frequency bands. Secondly, we will explore possible improvements of the confidence limits estimation, for example by splitting the time series in smaller windows, compute the confidence limits for each window, and then average the results (conceptually similar to the Welch’s periodogram approach), in order to reduce the variability of the average estimate. Thirdly, we expect to apply our approach to existing databases of HRV time series comparing healthy and pathologic patients, in order to test the capability of performing objective diagnosis/assessment from individual recordings. Finally, we expect to apply the MC and BS methods to other non-biomedical engineering applications, specifically fault detection of mechanical systems (e.g. bearings), which can be based on the changes of peaks locations and amplitudes in the vibration spectrum of rotating machines [32].

## Conclusions

By proposing empirical approaches for detecting confidence limits of quantitative measures derived from the autoregressive analysis of individual time series, the present study contributes to laying the groundwork for investigations focused on detecting properties which are peculiar to individual subjects, rather than a group to which they may belong. The proposed methods provide a statistical means to disentangle random errors in estimates from individual recordings from distinctive and significant features in the assessment of the differences between two measurements. They thus represent an additional statistical tool for the increasing number of studies directed towards achieving “personalized” approaches to medicine and physiology and phenotyping individuals in health and disease. The proposed tools are useful to assess inter-individual differences within a cohort otherwise considered “homogeneous”, or the subject-by-subject significance of changes of an index between different physiological conditions or stimuli. The great flexibility of the empirical approaches proposed in this study makes them suitable to the investigation of confidence limits for a broad range of measures derived from the parametric modelling of time series, and to be extended to measures derived from modelling univariate or multivariate systems in many biomedical applications and beyond.

## Supporting information

S1 File ARres—Matlab tool for computing confidence limits of measures derived from autoregressive modeling of time series. The toolbox provides Matlab functions to compute several resampled realizations of the AR parameters (first estimated using least squares (idAR.m)) based on Monte Carlo resampling (idARres.m) or bootstrapping of the AR residuals (idARboot.m, to be used with the built-in function bootstrp.m), from which Confidence limits for AR-based indexes can be computed from individual time series. Examples of indexes to which the procedures are applied are measures of frequency and spectral power obtained from AR spectral decomposition (asd_ARMASpectDec.m), and measures of information storage or complexity obtained from information decomposition (its_CElinVAR1.m). The toolbox is demonstrated on simulations of a fifth-order AR process resembling the spectral properties of heart rate variability time series (SimuConfLim.m).
(ZIP)

S2 File Database of R-R time series of 25 healthy subjects performing three psychophysiological tasks (rest, math silent, math aloud). This data is described and used in the section of the manuscript "Assessment of Methods using data recorded from human volunteers". The R-R time series are contained in the Matlab file RR_time_series.mat. The file contains 3 variables (rest, math_silent, math_aloud), each containing the R-R time series of all 25 subjects of the cohort for the respective task. Usage example: the R-R time series of subject 5 during the "math silent" task can be accessed as the 5th element of the cell variable math_silent (e.g. RR = math_silent{5};).
(ZIP)
